# bZIP Transcription Factors: Structure, Modification, Abiotic Stress Responses and Application in Plant Improvement

**DOI:** 10.3390/plants13152058

**Published:** 2024-07-25

**Authors:** Zhonglong Guo, Raphael Dzinyela, Liming Yang, Delight Hwarari

**Affiliations:** State Key Laboratory of Tree Genetics and Breeding, College of Life Sciences, Nanjing Forestry University, Nanjing 213007, China; guozhl@njfu.edu.cn (Z.G.); dzinyelar@njfu.edu.cn (R.D.); yangliming@njfu.edu.cn (L.Y.)

**Keywords:** bZIP, abiotic stress, modification, application

## Abstract

Plant growth, yield, and distribution are significantly impacted by abiotic stresses, affecting global ecosystems and forestry practices. However, plants have evolved complex adaptation mechanisms governed by numerous genes and transcription factors (TFs) to manage these stresses. Among these, bZIP (basic leucine zipper) is a crucial regulator orchestrating morphological adaptations. This review aims to elucidate the multifaceted roles of bZIP TFs in plant species. We discuss the morphological changes induced by stress stimuli and the pivotal functions of bZIP TFs in mediating these responses. While several publications have explored the mechanisms of bZIP TFs in response to abiotic stresses, this review delves into the intricate regulatory networks, summarizing alternative splicing and post-translational modifications, signaling networks interacting with bZIP TFs, and genetic engineering of bZIP TFs. By synthesizing current research, this review provides an updated discussion on bZIP interactions with other proteins to regulate stresses such as cold, heat, drought, and salt. Additionally, it offers avenues for future research and applications of bZIP TFs to improve abiotic stress resilience in plants through genetic engineering.

## 1. Introduction

Plant transcription factors (TFs) are crucial for cellular functions, dictating the roles and identity of cells. TFs bind specific DNA elements and recruit additional proteins for intrinsic biological processes [1]. Advances in molecular biology and biochemistry have led to the identification and elucidation of numerous TFs, including the basic region/leucine zipper motif (bZIP) TFs. The bZIP TFs are well-studied and named for their highly conserved bZIP domain, which consists of two structural features within the α-helix [2]. Phylogenetic studies have classified 78 bZIP TFs in *Arabidopsis thaliana* into 13 (A to M) groups based on the similarity of protein sequences, domain structures, and functionality [3]. For instance, gene members in Group A contain an N-terminal acidic region, which is absent in Group B bZIP factors. The classification of bZIP TFs is complex and evolves as new members are identified in other plant species. The bZIP TFs have also been classified based on functionality, such as stress response, developmental regulation, and metabolic regulation [4,5].

Recent studies have shown that bZIP TFs undergo several post-translational modifications (PTMs) that regulate their activity and stability, thereby affecting plant responses to both intra- and extracellular stimuli [6]. Detailed research has demonstrated that bZIP factors act as trans-acting factors that bind to the ABA-responsive element (ABRE; PyACGTGGC), with ABA signal transduction modified by several PTMs, such as phosphorylation, SUMOylation, and ubiquitination [7]. SnRK2-type protein kinases and calcium-dependent protein kinases phosphorylate bZIP TFs for their activation and stabilization [8]. Ubiquitination involves the degradation of bZIP factors through the ubiquitin-26S proteasome pathway, maintaining protein stability and transcriptional level. SUMOylation affects bZIP protein stability, activation, and localization, impacting plant stress and defense responses [9].

Genetic engineering of bZIP TFs has provided solutions for improving plant traits, enhancing stress tolerance, and increasing yield and nutritional content. Strategies employed include the overexpression of stress-responsive bZIP factors, which have alleviated environmental stresses such as drought, salinity, cold, and heat [10,11]. CRISPR/Cas9-mediated gene editing is another technique used to edit DNA sequences, encoding bZIP TFs through targeted mutations, deletions, or insertions [12,13]. Although some strategies still face challenges and drawbacks, genetic engineering of bZIP TFs holds great potential for enhancing plant resilience, productivity, and quality in response to environmental stresses and global climate changes.

This review aims to provide a comprehensive outline of bZIP TFs, highlighting their structural features, roles in abiotic stress responses, alternative splicing, post-translational modifications, and applications. Furthermore, this paper presents perspectives and challenges for research and applications of bZIP TFs, seeking to bridge the gap in the understanding of bZIP-mediated gene regulation and inspire future directions.

## 2. Structure of bZIP TFs

**Figure 1:**
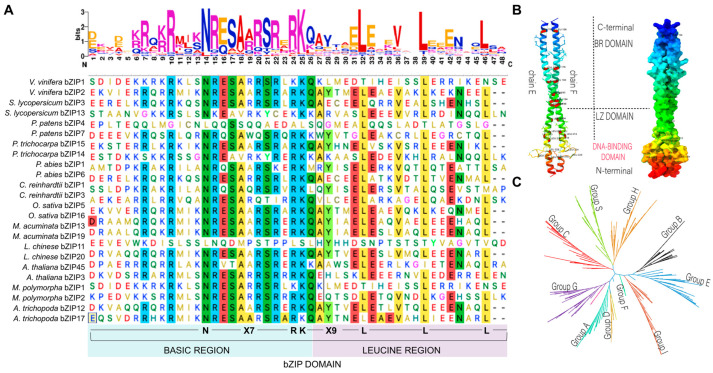
Structure and classification of bZIP. (**A**) Sequence alignment of bZIP domain from 12 plant species, highlighting the fixed nuclear localization structure N-x7-R/K-x9. The domain is divided into the basic region and the leucine zipper domain. (**B**) Three-dimensional protein structures of *Arabidopsis thaliana* bZIP4. The left figure illustrates the flat bZIP strands assigned as chains E and F, with leucine residues labeled and their position numbers included. The right 3D protein structure depicts the globular form of AtbZIP4, highlighting the DNA-binding domain. (**C**) Phylogenetic tree of bZIP from four plant species, including *Arabidopsis thaliana*, *Liriodendron chinense*, *Physcomitrella patens*, and *Oryza sativa*. The phylogenetic classification was generated using MEGA (version 11) software with default parameters and a bootstrap value of 1000.

bZIP TFs are highly conserved and have been identified in numerous plant species, including *Capsicum annum* [2], *Liriodendron chinense* [14], and *Populus trichocarpa* [15]. Structurally, bZIP TFs consist of a conserved domain of 60–80 amino acids, divided into the basic region and the leucine zipper domain [16]. The basic region, which is relatively conserved, consists of about 20 amino acids (Figure 1A). This region conforms to a fixed nuclear localization structure, N-x7-R/K-X9, which specifically binds to DNA cis-elements near its N-terminal (Figure 1A). It encompasses a stretch of positively charged amino acids, typically lysine (K) and arginine (R), facilitating DNA binding and recognizing bZIP binding elements or motifs such as 5′-TAC/GTCA-3′. The basic region also forms electrostatic interactions with the negatively charged phosphate backbone of the DNA, allowing binding to target genes.

In contrast to the basic region, the leucine zipper region is less conserved and contains two α-helices. Additionally, every sixth amino acid residue contains one leucine (Leu), or another hydrophobic residue such as isoleucine, valine, or methionine, forming a structure characterized by L-X6-L-X6-L. The leucine zipper, located in the C-terminal region, forms a coil structure. Two bZIP monomers dimerize through the interaction of their leucine zipper regions, creating a stable protein complex essential for the transcriptional activities of bZIP TFs (Figure 1B). The N-terminal of the zipper region associates with the acid domain to form either homologous or heterologous dimers responsible for transcriptional inhibition or activation.

bZIP TFs also contain several other sequence patterns, including glutamine-rich, proline-rich, and acidic-rich regions [17]. Many bZIP TFs have a transactivation domain in the N-terminal region, which is crucial for recruiting co-activators or co-repressors, thereby regulating transcriptional activity. This interaction also involves components of the transcriptional machinery, such as RNA polymerase II and other TFs. Research has shown that some bZIP TFs contain intrinsically discorded regions (IDRs) lacking stable structures or tertiary structures under physiological conditions. IDRs provide flexibility, allowing bZIP TFs to undergo conformational changes when binding to DNA or interacting with proteins. Additionally, IDRs serve as sites for PTMs, affecting the stability, DNA binding affinity, localization, and transcriptional activities of bZIP TFs.

As canonical trans-acting factors, bZIP TFs specifically bind to cis-acting elements in the promoter region of target genes, thereby activating or silencing their expression with temporal or spatial specificity [18]. The basic region of bZIP TFs preferentially binds to palindromic or non-palindromic cis-acting elements with an ACGT core, such as TACGTA (A-box), CACGTC (C-box), CACGTG (G-box), PB-like (TGAAAA), GLM (GTGAGTCAT), and ABRE (CCACGTGG). Some bZIP TFs also bind to other cis-acting elements, such as BRS1 (GTGCG), Dof (AAAG), I-box (GATAA), BS1 (AGCGGG), and MY3 (CGACG) [19], suggesting their involvement in response to different abiotic stresses.

The classification of bZIP TFs is complex due to the identification of new bZIP TFs in various plants. Nonetheless, all classifications agree on the division of bZIP TFs in plants based on specific amino acids, abundances, DNA binding sites, sequence conservation, and phylogenetic relationships (Figure 1C). For instance, in *Brassica rapa*, bZIP classification is based on amino acid abundances in nine groups, including glutamine (Q), aspartic acid (D), proline (P), asparagine (N), serine (S), glycine (G)-rich domains, transmembrane domain (Tm), and presence of low-complexity regions (LCRs).

## 3. Alternative Splicing and Post-Translational Modification of bZIPs

### 3.1. Alternative Splicing of bZIPs

Alternative splicing is vital in regulating gene expression and generating diverse protein structures for plant adaptation or development [20]. Changes in protein structure play an essential role in altering tissue specificity and enhancing protein binding, localization, and interactions. Thus, alternative splicing of mRNA produces several gene products with different biological functions [21]. Alternative splicing also links to abscisic acid (ABA) signaling by interacting with ABA-responsive cis-acting elements (ABRE), thereby regulating abiotic stresses by triggering the expression of stress-responsive genes [22]. For instance, studies in rice isolated two OsABI5 cDNAs, showing that both share an identical bZIP domain but differ by an extra 10 amino acid region behind the leucine zipper of OsABI5-2. Functional analysis revealed that OsABI5-1 and OsABI5-2 regulate different downstream genes [23,24]. Recently, the isolation of CiFD in *Citrus* showed that alternative splicing formed two proteins (CiFDα and CiFDβ) with similar expression patterns but different subcellular localizations and transcriptional activities. Both proteins are induced by drought and low-temperature stresses, although CiFDα with the florigen activation complex (FAC). Ci14-3-3 binds the CiAPETALA1 promoter for expression, while CiFDβ regulates drought stress independently of the FAC [25].

### 3.2. Post-Translational Modification of bZIPs

**Figure 2:**
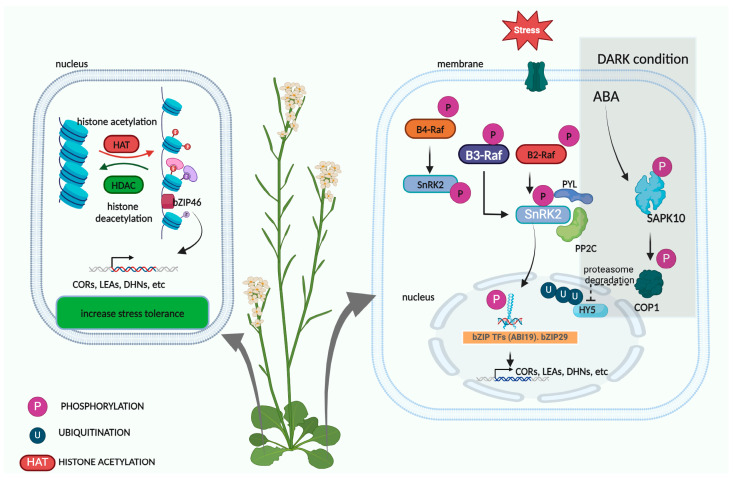
Post-translational modifications of bZIP TFs. The left panel illustrates the acetylation of bZIP TFs mediated by histone acetyltransferases and the deacetylation of bZIP TFs by histone deacetylases. Specifically, OsbZIP46 deacetylation mediated by the histone deacetylase OsHDA716 reduces chilling tolerance through the interplay between the chromatin regulator and the bZIP TF. The right panel depicts the phosphorylation and ubiquitination of bZIP TFs. SnRK2s mediate the ABA key signaling pathway to control transcription and stomatal aperture modulation. SnRK2.2 is responsible for ABA-induced phosphorylation at the threonine (T) site, while Raf-like protein kinases, including ABA and the ARK, act as upstream regulators of the SnRK2. During ubiquitination, the HY5 in *Arabidopsis* interacts with COP1 through phosphorylation reactions, leading to proteasome-mediated degradation and the addition of ubiquitin molecules to HY5.

Post-translational modifications (PTMs) are critical mechanisms in protein processing that regulate the activity, stability, localization, and protein-to-protein interactions of bZIP TFs. PTMs include phosphorylation, SUMOylation, acetylation, ubiquitination, and more. Collectively, these PTMs offer complex regulatory mechanisms that fine-tune the activity and function of bZIP transcription factors in response to cellular signals and abiotic stresses (Figure 2).

Phosphorylation plays a pivotal role in regulating bZIP TFs, occurring on serine, threonine, or tyrosine residues within the bZIP domain or other regions. Research has demonstrated that phosphorylation of bZIP TFs affects their DNA-binding affinity and subcellular localization [26]. Several kinases and phosphatases facilitate the phosphorylation and dephosphorylation processes in response to extracellular signals and stress stimuli. Phosphorylation by protein kinases is critical in ABA signaling and drought stress responses. The most studied protein kinases involved in drought stress regulation are SnRK2-type kinases (SnRK2s) and receptor-like kinases (RLKs). SnRK2s mediate key ABA signaling pathways to control transcription and stomatal aperture modulation, while RLKs transmit signals to downstream target proteins through sequential phosphorylation processes. For example, SnRK2.2 is responsible for the ABA-induced phosphorylation of threonine (T) 57 in ZmABI19, T75 in ZmbZIP29, and T387 in Opaque 2, essential for maximum transactivation of downstream target genes [27].

Raf-like kinases (ARK) act as upstream regulators of the subgroup III SNF1-related protein kinase (SnRK2s), regulating ABA and abiotic stress responses [28] (Figure 2). SAPK10, a member of SnRK2, interacts with OsbZIP20 through phosphorylation. A phos-tag method revealed that OsbZIP20 carries phosphorites at the sixth serine (Ser) on their amino acid sequences, requiring the phosphorylation activities of OsSAPK10 for regulatory roles [19]. ELONGATED HYPOCOTYL 5 (HY5), a bZIP TF in *Arabidopsis*, interacts with CONSTITUTIVELY PHOTOMORPHOGENIC1 (COP1) through phosphorylation reactions and reduces contents by proteasome-mediated degradation (Figure 2) [29]. However, light stress negatively regulates COP1 levels, reducing its accumulation and increasing phosphorylated HY5 levels necessary for dark-to-light transitions [29].

Acetylation of bZIP transcription factors involves the addition of acetyl groups to lysine residues through the action of histone acetyltransferases (HATs) (Figure 2). Like phosphorylation, acetylation modulates stability, DNA-binding affinity, transcriptional activity, and protein-to-protein interactions of bZIP TFs [30]. Conversely, deacetylation of bZIP TFs by histone deacetylases (HDACs) reverses acetylation, serving as a regulatory measure to govern bZIP TF activity and abundance. In rice, OsbZIP46 deacetylation mediated by histone deacetylase OsHDA716 reduced chilling tolerance through the interplay between the chromatin regulator and bZIP transcription factor [31] (Figure 2). Knockout of HDA710/OsHDAC2 in rice induced transcription activation of OsbZIP72, increasing ABA- and salt-stress-responsive genes by stabilizing H4 acetylation levels in promoter regions [32]. Histon acetylation of ABI5, a bZIP TF, by H3K9Ac through regulation of a histone-binding protein, ENAP1, in promoter regions increased the levels of ABI5 and consequently seed germination, suggesting that H3K9Ac is a negative regulator of ABI5 for inhibiting seed germination [33].

Ubiquitin molecules covalently attach to lysine residues of bZIP TFs, marking them for proteasome degradation. Ubiquitination regulates the protein yield, abundance, and activity of bZIP TFs, influencing gene expression and cellular responses to external stimuli. HY5, a bZIP TF, is a master regulator of de-etiolation and photomorphogenesis, regulated by the E3 ubiquitin ligase complex composed of COP1 and SUPPRESSOR OF PHYA-105 (SPA) proteins (COP1/SPA) (Figure 2). Research suggests that COP1/SPA mediates HY5 degradation in the dark, allowing plants to quickly switch from etiolated to de-etiolated growth upon light perception [34]. COP1 is a negative regulator of HY5. However, low temperatures trigger COP1 translocation from the nucleus to the cytoplasm [35]. PREFOLDIN 4 (PFD4), readily available in the nucleus, interacts with HY5 to facilitate its polyubiquitination and degradation in a COP1-independent manner under low-temperature stress [36]. Phosphorylated blue light photoreceptor CRY2 competes with COP1 in interacting with HY5, increasing HY5 levels and consequently activating transcription of BBX7 and BBX8. Research has established that BBX7 and BBX8 induce transcription of various cold-responsive genes that promote cold stress tolerance [37], including MYB15 and CBF1-3 in the tomato [38], and MYB108 in the CBF-cold responsive pathway [38].

## 4. Regulation of Plant Response to Abiotic Stresses

### 4.1. Interact with Stress-Responsive Proteins

**Figure 3:**
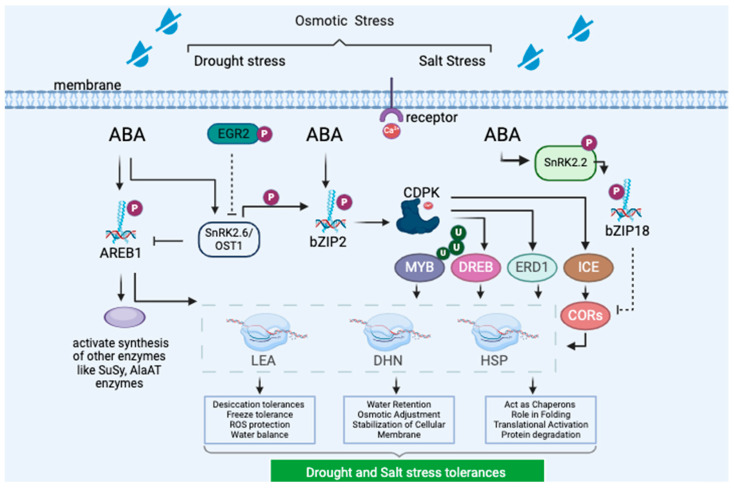
Interaction of bZIP TFs with other proteins to regulate drought and salt stresses. AREB1 induces the transcription of drought-responsive genes such as LEA18, dehydrin, and HSP70 through ABA-mediated activation. Additionally, AREB1 induces the transcription of enzymes like SuSy and genes encoding AlaAT enzymes. bZIP2 TFs binds to ACGT elements in the promoter regions of MYB48, WD40, DHN15, and LEA to regulate salt and drought stresses.

Cold stress responsiveness is critical for plant adaptation to changing environments. bZIP TFs activate cold stress-responsive genes, which typically encode proteins involved in cold tolerance, such as antifreeze proteins, cold shock proteins, dehydrins, and enzymes critical for osmoprotectant synthesis [39]. Among these, the bZIP AREB/ABF genes, including AREB1/ABF1, AREB2/ABF4, and ABF3, confer resistance to drought stress by interacting with drought-responsive genes like LEA, dehydrins, and HSPs. Late Embryogenesis Abundant (LEA) genes encode proteins expressed during the late stages of seed development and play roles in desiccation tolerance, osmoprotection, freeze tolerance, and ROS protection. Dehydrins help retain water, protect against ROS, stabilize protein-membrane structures, and regulate gene expression and signaling pathways [39]. HSP genes act as molecular chaperones, assisting in protein folding, translocation, and degradation under stress (Figure 3).

bZIP TFs bind to cis-regulatory elements in the promoter regions of these genes, activating their transcription in response to cold stress signals. For instance, in tomatoes, SIAREB1 bZIP TF expression is induced by cold stress through the ABA-mediated signaling pathway, coupled with transcription activation of SnRK2.6/OST1. Overexpression of SIAREB1 showed it binds to the promoter regions of SICI7-like dehydrin and SILAP to regulate cold stress [40]. Similarly, overexpressed TaABI5 (TabZIP96) interacts with TaICE1 and regulates cold stress by increasing CAT and POD activities, reducing ROS accumulation, and suggesting regulation through the ICE-CBF-COR pathway [41,42]. Additionally, studies have shown that ABI5 interacts with other cold-responsive genes, such as COR15A, COR6.6, and RAB18, to regulate cold stress [43]. However, some bZIP TFs negatively regulate cold stress. In rice, OsbZIP52 increased sensitivity to cold and drought by downregulating stress-responsive genes like OsLEA3 and OsRab25, binding to G-box motifs in promoter regions to inhibit transcription [44] (Figure 3, Table 1).

In *Arabidopsis*, overexpressed AtAREB1 induces the transcription of drought-responsive genes like LEA18, dehydrin, and HSP70 through ABA activation. AtAREB1 also increases drought stress tolerance by controlling ROS and protein accumulation [57]. In *Glycine max*, overexpressed bZIP2 increases drought and salt tolerance by activating transcription of GmMYB48, GmWD40, GmDHN15, and GmLEA [53]. Maize bZIP TFs interact with HsF08 to regulate salt and drought stress, activating ZmDREB2A, ZmNCED, ZmERD1, ZmRD20, and ZmRAB18 [58]. In wheat, Ser-110 within the C2-C3 domain of TabZIP60 interacts with TaCDPK30 to regulate salt stress through ABA-mediated pathways, increasing soluble sugar levels, MDA content, and transcription of stress-responsive genes like TaLEA6-3 and TaWZY2 [59]. Another study showed that TabZIP60 interacts with TaCDPK5 and TaCDPK9-1, thereby regulating abiotic stresses [60].

### 4.2. Interaction of bZIPs with Hormonal and MAPK Signaling Pathways

#### 4.2.1. Interaction of bZIPs with Hormonal Signaling Pathways

bZIP TFs interact with hormonal pathways to regulate plant responses to environmental cues, involving phytohormones such as abscisic acid (ABA), ethylene (ET), jasmonic acid (JA), salicylic acid (SA), and gibberellins (GA) [61,62]. Ethylene is a central phytohormone for plant stress response, growth, and development. bZIP TFs interact with ethylene to modulate gene expression under abiotic stress conditions. For instance, blue light treatment has been shown to inhibit ethylene production in pear, essential for fruit ripening. Additional investigations revealed that HY5 binds to ACC synthase 1 (PuACS1), a rate-limiting enzyme in ethylene biosynthesis, inhibiting its expression. This indicates that PpHY5 mediates ethylene synthesis under blue light stress [63]. Light-emitting diode (LED) white light was also shown to induce the transcription of two bZIP genes, MdHY5L and MdHY5S, in *Malus domestica*. Further investigation revealed that MdHY5L and MdHY5S inhibited the transcription of ACS1, an ethylene biosynthesis gene, delaying ethylene production. Analysis showed that MdHY5L and MdHY5S bind to the hybrid CG motif (GACGTG), inhibiting ACS1 gene expression [64]. In other studies, MabZIP21 was observed to interact with MaWRKY49 and MaWRTKY111, synergistically enhancing the transcription of MaACS1 and MaACO1 and increasing ethylene production [65]. In *Prunus persica*, cold stress increased ethylene production and the transcription of Prupe.6G343100, a homolog of AtbZIP25. It was speculated that Prupe.6G343100 regulates ACS1 by binding to its ABRE promoter region [66]. Long-term low-temperature storage of peaches induces ethylene and ABA production, coupled with the downregulation of PpbZIP23 and PpbZIP25, regulated by the ABA-induced cold induction pathway [67].

Abscisic acid (ABA) is crucial for regulating plant stress, particularly drought and osmotic stress [68]. Its roles include stomatal closure, osmotic stress tolerance, and induction of stress-responsive genes. bZIP transcription factors are involved in ABA signaling and response [69]. bZIP TFs, such as ABRE-binding factors or ABA-responsive element binding proteins (ABREs), regulate ABA-responsive genes by binding to ABRE elements in their promoter regions. Overexpression of *Camellia sinensis* bZIP18 in *Arabidopsis* acts as a negative regulator of cold stress, inhibiting the expression of cold-response genes such as AytCOR, AtRD22/26, and AtRAB18, induced in the ABA-independent CBF pathway. CsbZIP18 participates in the ABA-signaling pathway by activating upstream kinases like SnRK2s through phosphorylation [70].

Jasmonic acid and salicylic acid also interact with bZIP TFs in plant defense against abiotic stress. Through these interactions, bZIP factors regulate the expression of stress-responsive genes and enhance tolerance to abiotic stresses. Overexpression of bZIP19 in *G. max* showed downregulation of ABA-, JA-, ETH- (ethephon), and SA-induced marker genes, leading to salt and drought stress sensitivity. Chromatin immunoprecipitation assays disclosed that GmbZIP19 binds to these marker genes, inhibiting their expression, indicating that GmbZIP19 is a negative regulator of salt and drought stresses [52]. The Jasmonate ZIM-domain (JAZ) protein in the JA pathway is involved in environmental defense responses. Expression profiles of JAZ genes in Helianthus annus L. under hormonal and abiotic stress demonstrated that HaJAZ5/15/17/29/21 are sensitive to cold, salt, and drought stresses, with further investigations suggesting that bZIP TFs may play a role in the transcription activation of HaJAZs [71]. In *Medicago truncatula*, bZIP17 and bZIP60, localized in the nucleus, interfere with ER stress-related and JA-modulated bHLH TFs in regulating JA-dependent terpene biosynthesis [72].

#### 4.2.2. Interaction of bZIPs with MAPK Signaling Pathways

Abiotic stresses such as drought and salt trigger intricate signaling networks involving several protein kinases and transcription factors, including interactions with the MAPK cascade and SnRK2 (Sucrose non-fermenting 1-related protein kinase 2). These signaling networks activate bZIP TFs, leading to the transcription of stress-responsive genes involved in osmoprotection, water balance, and stress alleviation. For instance, Stress/ABA-activated Protein Kinase 2 (SAPK2), homologous to SnRK2, interacts with phosphorylated OsbZIP23 for its transcriptional activation, regulating drought stress through ABA signaling. Additional studies revealed that SAPK2 interacts with OsPP3C49, another homolog of ABI1, to inhibit OsbZIP23 transcription [73].

Furthermore, bZIP TFs and the MAPK cascade interact to regulate crucial mechanisms involved in abiotic stress responses. Research has shown that the MAPK cascade is a conserved signaling entity critical in transmitting extracellular signals to the nucleus and modulating gene expression and cellular responses. Abiotic stresses like drought, cold, salinity, and heat activate MAPKKKs (MAP kinase kinase kinase), which then phosphorylate and activate downstream MAPKKs (MAP kinase kinase), leading to the phosphorylation and activation of MAPKs. Activated MAPKs phosphorylate various transcription factors, including bZIP TFs, which modulate their activity, stability, and subcellular localization, ultimately affecting their interaction with other proteins. MAPK-phosphorylated bZIP TFs regulate the expression of stress-responsive genes by binding to their promoter regions, enabling the activation of these genes and increasing abiotic stress tolerance. For example, in banana, bZIP74 interacts with MaMAK11-3, a protein kinase, through phosphorylation, attenuating MabZIP74-mediated transcriptional repression of MaCO1 and MaCO4, which are involved in ethylene production [74].

The bZIP TF activated and phosphorylated by the MAPK cascade can integrate various stress signals from different pathways, as the MAPK cascade relays signals from diverse upstream receptors and sensors to bZIP TFs, coordinating the transcriptional response to different abiotic stresses. This integration enables plants to optimize their adaptive responses based on stress severity and duration. Moreover, the MAPK cascades cross-talk with other signaling pathways, such as ABA and ROS signaling, and other phytohormone pathways, further enhancing the regulatory mechanisms governing abiotic stress responses. Therefore, bZIP TFs in the MAPK cascade signaling pathway act as key nodes integrating and transmitting signals to downstream target genes for stress tolerance.

## 5. Genetic Engineering of bZIP TFs

Genetic engineering of bZIP TFs offers significant possibilities for modulating gene expression and improving plant traits, particularly in response to abiotic stresses [75]. Common strategies include genome-wide identification and functional characterization, overexpression, CRISPR/Cas-mediated gene editing, and the creation of chimeric TFs. Numerous studies have focused on the genome-wide analysis and functional characterization of bZIP TFs. These studies systematically identified genes in the bZIP TF family, providing insights into their functional roles through evolutionary relationships, conservation of the bZIP domain and motifs, protein-to-protein interaction assays, and phenotypic analyses. Several bZIP TFs have been identified in pepper [2], wheat [56], tobacco [76], and poplar [15]. These studies provide a solid foundation for further exploration of gene functions.

Overexpression of specific bZIP TFs in plants enhances their transcriptional activity, altering the expression of target genes and subsequently improving tolerances to drought, cold, heat, and salt [77]. For example, the salt-induced bZIP gene, TabZIP15, was overexpressed in maize and wheat, resulting in increased salt tolerance. Further study suggested that TabZIP15 interacts with TaEnNO-b, an enolase protein that catalyzes the reversible dehydration of 2-phospho-D-glycerate to phosphoenolpyruvate in glycolytic and gluconeogenesis pathways [56]. Similarly, overexpression of TubZIP28 in wheat increased starch content by binding to the promoter of cytosolic AGPase, enhancing both its transcription and activity [78]. In tobacco, overexpression of NtbZIP62, a homolog of AtbZIP37/ABF3, significantly enhanced salt tolerance in transgenic lines [79].

CRISPR/Cas-mediated gene editing has revolutionized plant genomics by introducing targeted mutations, deletions, and insertions in bZIP genes, manipulating their protein function and expression levels [75]. The CRISPR/Cas9 technique, derived from the Streptococcus pyogenes type II CRISPR system (spCas9), involves two noncoding RNAs: a trans-activating RNA (tracr-RNA) and a long pre-CRISPR RNA (pre-crRNA), along with a single Cas9 nuclease [80,81]. The pre-crRNA guides the Cas9 nuclease, which binds and cleaves DNA through the activities of sgRNA and a protospacer adjacent motif (PAM), creating double-strand breaks (DSB). The DSBs are repaired through either non-homologous end joining (NHEJ) or homology-directed repair (HDR). HDR prompts deletions or inserts bases at the targeted site, while NHEJ generates new alleles. The CRISPR/Cas9 technique allows for precise editing of DNA sequences encoding bZIP TFs. For instance, Song et al. used CRISPR/Cas9 to knock out TabZIP28 in wheat, demonstrating that TabZIP28 is a negative regulator of cytosolic AGPase transcription and activation in developing endosperms [78]. In *Actinidia eriantha*, knockout of PosF21, a bZIP TF, decreased L-ascorbic acid (Asa, vitamin C) synthesis and increased ROS generation, thereby regulating cold stress through the AcePosF21-AsA biosynthesis pathway [82]. Gene knockout of PtrbZIP3 using CRISPR/Cas9 demonstrated its role in drought stress regulation by lowering toxic substance production and protecting the cell membrane from damage [45]. In rice, knockout of OsbZIP72 revealed its function in resisting drought and salt stresses. Overexpression study revealed that OsbZIP72 binds to the ABA-responsive element in the promoter of OsHKT1;1, activating its transcription to regulate salt and drought stresses [83].

Other genetic engineering techniques used to enhance bZIP TFs include promoter engineering and creating chimeric transcription factors. Promoter engineering involves modifying the promoter regions of bZIP genes to influence their expression patterns and levels in response to specific abiotic stress stimuli. Chimeric TFs are created by fusing the DNA-binding domain of bZIP factors with transcriptional activation or repression domains from other regulatory proteins to modulate the activity of target genes. For example, chimeric HY5 variants were generated by adding a transcriptional silencing motif (EAR repressor motif of *Arabidopsis* SUPERMAN gene [SRDX]) or the activation domain from V16 to determine its transcriptional regulations. These HY5 variants were expressed under the control of the constitutive 35S promoter, showing rapid upregulation in response to high temperatures [34].

## 6. Future Perspectives, and Challenges

The bZIP TFs play a crucial role in regulating stress-responsive genes and coordinating cellular mechanisms that govern plant responses to environmental cues. Their modular structures, DNA-binding affinity, dimerization capability, and ability to interact with other proteins equip bZIP TFs to function effectively in stress tolerance and adaptation. In this review, we have highlighted several alternative splicing and post-translational modifications involved in regulating bZIP TFs, demonstrating how these modifications determine stability, localization, transcriptional activity, structure, and DNA-binding affinity.

Plants often encounter multiple stresses simultaneously, posing challenges for dissecting the individual and combined effects of different stressors on bZIP TF regulation and function. Integrating multi-omics approaches and computational modeling will be crucial for unraveling the complexity of stress signaling networks in plants. Some bZIP TFs exhibit functional redundancy or overlapping roles, making it challenging to elucidate their specific functions and regulatory mechanisms. Disentangling the complexity of bZIP TF networks and deciphering their precise roles in stress responses require sophisticated genetic and genomic approaches. Further research is needed to dissect the regulatory networks controlled by bZIP TFs and to deepen our understanding of how these factors integrate with various stress signal pathways to regulate gene expression. Harnessing the regulatory roles of bZIP TFs could facilitate the development of crop plants with enhanced stress tolerance and productivity. Targeted manipulation of bZIP TFs through genetic engineering or breeding strategies holds great promise for improving agricultural resilience to climate change and environmental stresses.

In summary, bZIP TFs represent promising targets for understanding and engineering plant stress responses. Continued research efforts aimed at unraveling their regulatory mechanisms, exploring their potential for crop improvement, and addressing existing challenges will contribute to enhancing agricultural sustainability and food security in a changing climate. Genetic engineering of bZIP transcription factors holds significant promise for enhancing crop resilience, productivity, and nutritional quality in the face of environmental challenges and changing climate conditions. However, it is essential to consider potential off-target effects, regulatory constraints, and ethical considerations associated with the release of genetically modified crops into the environment. Collaborative efforts between researchers, breeders, regulators, and stakeholders are crucial for the responsible and sustainable deployment of genetically engineered bZIP factors in agriculture.

## Figures and Tables

**Table 1:** A summary of bZIP TFs in response to abiotic stresses.

Plant	Abiotic Stress	bZIP Gene	Target Downstream Genes/Pathway	Cite
*Populus trichocarpa*	Drought and salinity	*PtrbZIP3*	ABA-dependent signaling	[45]
*Zea mays*	Drought and heat	*ZmNF-YA3*	bHLH92, FAMA, and MYC4	[46]
	Salt and osmotic	*ZmbZIP76*	ABA-dependent signaling, ZmNCE3/5, ZmCOR47, and ZmRD29A	[47]
*Oryza sativa*	Drought and oxidative	*OsbZIP62*	OsCL1, OsNAC10, OsDSM2, and OsSAPKs	[48]
	Drought and yield performances	*OsbZIP63*	OsRab16B, OsRab21, and OsLEA3-1	[49]
	Drought	*OsbZIP42*	OsLEA3, OsRab16 and ABA-dependent signaling.	[50]
	Drought	*OsABF1*	OsCRR413-TM1, OsPP2Cs, and other OsbZIPs	[50]
	Salt and drought	*OsbZIP20*	OsSAPK10, OsNXH1	[19]
*Glycine max*	Salt and heavy metal	*GmbZIP152*	AtLOX6, AtACS, AtERF1, and AtABI2	[51]
	Drought stress sensitive	*GmbZIP19*	AtABI5, AtACS6, AtLOX4, AtERF1, AtPR1, and AtWRKY26	[52]
	Salt and drought	*GmbZIP15*	GmSAHH1, GmWRKY12, and GmABF1	[11]
	Salt and drought	*GmbZIP2*	GmMYB48, GmWD40, GmDHN15, GmGST1, and GmLEA	[53]
*Arabidopsis thaliana*	Drought, oxidative and nitro-oxidative	*AtbZIP62*	AtPYRD, AtCAT2, and JA signaling pathway	[54]
	Salt	*AtbZIP4*	ABA-signaling AtCaM1, AtRD22,	[55]
*Triticum aestivum*	Salt	*TabZIP15*	TaENO-b	[56]
*Capsicum annum*	Salt	*CabZIP25*	-	[2]

## Data Availability

The original contributions presented in the study are included in the article, further inquiries can be directed to the corresponding author.
